# Toll-Like Receptor Signaling Pathways: Novel Therapeutic Targets for Cerebrovascular Disorders

**DOI:** 10.3390/ijms22116153

**Published:** 2021-06-07

**Authors:** Rezan Ashayeri Ahmadabad, Zahra Mirzaasgari, Ali Gorji, Maryam Khaleghi Ghadiri

**Affiliations:** 1 Shefa Neuroscience Research Center, Khatam Alanbia Hospital, Tehran 1996835911, Iran; re.ashayeri@gmail.com (R.A.A.); mirzaasgari@gmail.com (Z.M.); 2 Department of Neurology, Iran University of Medical Sciences, Tehran 1593747811, Iran; 3 Epilepsy Research Center, Westfälische Wilhelms-Universität, 48149 Münster, Germany; 4 Neuroscience Research Center, Mashhad University of Medical Sciences, Mashhad 9177948564, Iran; 5 Department of Neurosurgery, Westfälische Wilhelms-Universität, 48149 Münster, Germany; Maryam.KhaleghiGhadiri@ukmuenster.de; 6 Department of Neurology, Westfälische Wilhelms-Universität Münster, 48149 Münster, Germany

**Keywords:** stroke, inflammatory mediators, chemokines, brain, cell injury

## Abstract

Toll-like receptors (TLRs), a class of pattern recognition proteins, play an integral role in the modulation of systemic inflammatory responses. Cerebrovascular diseases (CVDs) are a group of pathological conditions that temporarily or permanently affect the brain tissue mostly via the decrease of oxygen and glucose supply. TLRs have a critical role in the activation of inflammatory cascades following hypoxic-ischemic events and subsequently contribute to neuroprotective or detrimental effects of CVD-induced neuroinflammation. The TLR signaling pathway and downstream cascades trigger immune responses via the production and release of various inflammatory mediators. The present review describes the modulatory role of the TLR signaling pathway in the inflammatory responses developed following various CVDs and discusses the potential benefits of the modulation of different TLRs in the improvement of functional outcomes after brain ischemia.

## 1. Introduction

Cerebrovascular diseases (CVDs) are pathological conditions that cause a reduction in the blood supply to the brain [1]. The most common CVDs consist of ischemic disorders, such as acute ischemic stroke (AIS) and cerebral venous sinus thrombosis (CVST), as well as hemorrhagic conditions, like intracerebral hemorrhage (ICH) and subarachnoid hemorrhage (SAH) [2]. Neuroinflammation caused by hypoxia or hypoxic-ischemic insults is a key cellular pathway of cellular injury and plays a crucial role in the level of neural tissue damage and functional recovery [3,4,5,6,7]. CVDs can benefit from targeting ischemia-induced inflammation to prevent or decrease neuronal damages and/or promote neuroplasticity and recovery [8]. Following ischemic injury, inflammatory molecules are released from injured and dead cells, which provoke a local inflammation and exacerbate the ischemic condition [9,10]. The inflammation following hypoxic-ischemic insults is mainly characterized by the activation of resident microglia, the recruitment of peripheral macrophages and leucocytes to the nervous system, and the release of cytokines, chemokines, and reactive oxygen species [11,12]. The release of inflammatory mediators induces an activated state of leucocytes and cerebral endothelial cells [13]. Moreover, mediators released following ischemic insults promote the transcription of different genes, like tumor necrosis factor (TNF), nuclear factor kappa B (NF-kB), and Toll-like receptors (TLRs), which make a significant contribution to the regulation of the hypoxia-induced inflammatory responses [14,15]. TLRs, a class of pattern recognition receptors, are type I transmembrane proteins with a crucial role in the identification of pathogen molecules, like flagellin, zymosan, profilin-like proteins, microbial membrane components, and bacterial lipopolysaccharides [11,16]. TLRs detect proteins, lipids, and nucleic acids of a wide range of pathogens and are expressed by neurons, astrocytes, and oligodendrocytes as well as by antigen-presenting cells, including microglia, monocytes, macrophages, B cells, and dendritic cells within the central nervous system (CNS) [17,18,19]. Moreover, various cytokines like interferons, interleukins, and chemokines, which exist in the surrounding ischemic brain tissue, can also activate these receptors [20]. The interaction between TLRs and cytosolic pattern-recognition receptors, particularly RIG-I-like and Nod-like receptors, is critical for rising appropriate immune responses in the CNS [21].

Numerous amounts of evidence revealed that hypoxic-ischemic insults and neuroinflammation attribute to the pathogenic mechanisms of CVDs [22,23]. Increasing evidence suggests the crucial role of TLRs in the pathogenesis of AIS [24,25], ICH [26,27], and SAH [28]. Activation of TLRs following hypoxic-ischemic injury may either be protective or detrimental, depending on the context. Following a sublethal hypoxic-ischemic injury, activation of TLRs can cause a greater cerebral ischemic tolerance, a process called preconditioning. However, activation of TLRs after ischemia-induced tissue and cell injury can worsen tissue damage through the NF-kB signaling pathway [21,29,30]. TLRs and their downstream signaling molecules represent a relevant pharmacologic target for modulation of tolerance to an ischemic insult [31]. Modulation of TLRs alters the immune responses and ischemic tolerance in CVDs [32,33,34,35,36]. Among the various TLRs subtypes, those which may influence CVDs-induced tissue injury and their neurological deficit are mainly TLR2, TLR3, TLR4, TLR7, and TLR9 (Table 1). Moreover, it has been suggested that drugs that target TLRs, especially TLR3 and TLR4, exert neuroprotective effects against various CVDs [20,34,37].

## 2. General Aspects of TLR Signaling Pathways

**Figure 1:**
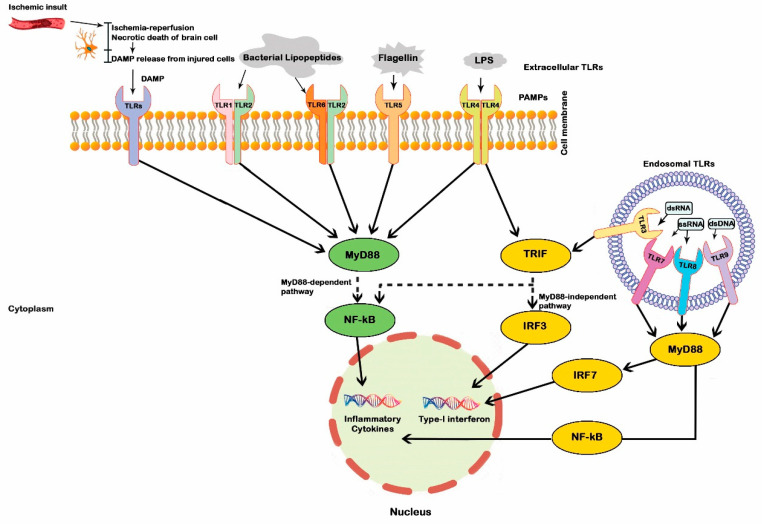
Diagrammatic representation of the Toll-like receptor (TLRs) signaling pathways. TLRs are expressed in neurons, microglia, astrocytes, oligodendrocytes as well as neural stem cells. DAMPs: Damage-associated molecular patterns; LPS: Lipopolysaccharide; dsDNA: Double-stranded DNA; dsRNA, Double-stranded RNA; ssRNA: Single-stranded RNA; MyD88: Myeloid differentiation primary response 88; PAMPs: Pathogen-associated molecular patterns; NF-kB: nuclear factor kappa B; IRF: Interferon regulatory factor; TRIF: TIR-domain-containing adapter inducing IFN-β.

TLRs have a crucial role in the innate and adaptive immune systems through their modulatory effects on various immune cells [38,39,40,41,42]. They are comprised of TLR1 through TLR13 [43]. TLRs express both on the cell surface and in the intracellular space (endosomal TLRs) [44]. TLR1, TLR2, TLR4, TLR5, TLR6, TLR10, and TLR11 are embodied on the cell membrane, and TLR3, TLR7, TLR8, and TLR9 are localized in the intracellular compartment (Figure 1) [45]. Different combinations of TLRs are widely expressed in various cells within the CNS, including microglia, neurons, astrocytes, oligodendrocytes, and neural stem/progenitor cells, as well as vascular endothelial cells and epithelial cells [14,46]. Activation of TLR-mediated signaling pathway regulates antigen uptake and presentation and immune cell maturation as well as cytokine production and release [47]. Binding of damage-associated molecular patterns and pathogen-associated molecular patterns, molecular motifs localized on pathogens, to TLRs and their conformational alterations stimulate a cascade of downstream signaling and trigger inflammatory responses [48]. TLR signaling is generally divided into two main downstream adaptor proteins; the myeloid differentiation primary response 88 (MyD88)-dependent and adapter-inducing interferon-β (TRIF)-dependent pathways [49]. The canonical adaptor molecule MyD88 activates NF-κB through nearly all TLRs, except TLR3 [50]. The main function of the TRIF-dependent TLR pathway is to activate NF-kB and interferon-regulatory factor (IRF), mainly via TLR3 and TLR4 [51]. The transcription factor NF-kB induces the production of various pro-inflammatory cytokines, such as TNFα, IL1β, IL6, IL8, and IL12, whereas IRF regulates transcription of type I interferons (IFN) and its antiviral activities [35]. TLRs can also modulate inflammatory responses via the regulation of AMP-activated protein kinase, a serine/threonine-protein kinase that acts on several metabolic pathways [52,53]. Moreover, neutrophil extracellular traps (NET) formation is associated with neural tissue hypoxia following traumatic brain injury (TBI). TLRs and the downstream kinase peptidyl-arginine deiminase 4 regulated NET formation, which contributes to hypoxia-induced tissue injury [54]. Moreover, high mobility group box 1 (HMGB1), a DNA-binding protein, has been shown to actively participate in inflammatory responses through binding to TLRs after CNS hypoxic events. HMGB1 is localized in cell nuclei and moves into the cytosol and then into the extracellular space after the hypoxic insults [55]. Extracellular HMGB1 mostly interacts with TLR2 and TLR4 to provoke inflammatory responses via the NF-kB pathway [56]. HMGB1 is a target for therapeutic approaches to various cancers and may play a role in the treatment of CVDs [57,58].

## 3. The Role of Toll-Like Receptors in CVDs

TLRs signaling cascades are involved in the pathophysiology of numerous CNS diseases. There is growing evidence of the implication of TLRs in various neurodegenerative disorders [59,60], including Alzheimer’s disease [61,62,63], Huntington’s disease [64,65], Parkinson’s disease [66], dementia [67], and amyotrophic lateral sclerosis [68,69]. Furthermore, TLRs implicated in demyelinating diseases, like multiple sclerosis [70,71] and optic neuritis [72] and acute neurological disorders, such as epilepsy [73] and migraine headache [74], as well as in CNS infections [75], and traumatic brain injury [76]. Moreover, TLRs play a role in the modulation of cognitive processes, including learning and memory, and are involved in the pathobiology of psychological disorders, such as anxiety [77], schizophrenia [78,79], depression [80]. Modulation of these receptors has been studied in different CVDs (Table 1).

### 3.1. The Role of TLRs in AIS

Endothelial cells play a pivotal role in the regulation of inflammatory procedures [118] and are the main contributor to vascular integrity [119]. Notably, Dysfunction of the arterial endothelial lining of hypoxic-ischemic brain tissues contributes to the pathophysiology of inflammatory responses in AIS [120,121]. Indeed, several TLRs, particularly TLR2, TLR4, and TLR9, are found in the endothelial cell membrane and involved in endothelial dysfunction as well as in the development and progression of atherothrombosis [121,122,123,124]. Various adhesion molecules on the endothelial cells regulate the transcription of TLRs by modulation of NF-kB levels [125].

Interestingly, the activation of TLRs after AIS exerts complex and biphasic effects, both beneficial and detrimental, on ischemia-induced tissue injury. A mild ischemic injury inhibited the TLR2 and TLR4/NF-kB signaling pathway and activated IRF3 signaling, which led to cortical neuroprotection in the acute phase of ischemic injury [126]. On the other hand, pharmacological preconditioning with TLR2, TLR3, TLR4, TLR7, or TLR9 agonists before the induction of ischemic brain injury improves neuroprotection and decreases the ischemic damage in various animal models [29,115]. For example, lipopolysaccharide (LPS), an outer membrane portion of gram-negative bacteria, triggers the innate immune response by the activation of TLR4 [127]. Moreover, LPS contributes to the development of cavernous angiomas, a vascular malformation that may cause AIS, via the TLR signaling pathway [128]. Several investigations indicate that the application of LPS before ischemia increases the tolerance of neuronal tissues to the hypoxic insults via TLR4 [115,129,130]. Indeed, the LPS-induced neuroprotective effect after AIS is through the modulation of the TLR4 signaling, inhibition of NF-kB activity, and enhancement of IRF3 activity and IFN expression [131]. In one study, the application of a single dose of LPS a week before ischemia induced by middle cerebral artery occlusion (MCAO) decreased the infarct volume in hypertensive rats [132]. In addition, adrenaline promotes macrophage response to the LPS stimulation and interacts with TLRs, which leads to the induction of a pro-inflammatory cascade [133,134]. Conversely, inhibition of β-adrenergic receptors reduces LPS-induced inflammatory responses via TLR4 [135]. For example, pretreatment with propranolol, a nonselective β-adrenergic receptor blocker reduces inflammation, cytokine production, hyperglycemia, infarction volume, edema, and apoptosis in rodent models of AIS [90,91,136,137]. Under other conditions, LPS can exert cytotoxic effects after AIS [138,139]. Enhancement of plasma LPS values increased the expression of TLR4, inflammatory cytokine levels, infarct volumes, and neurological deficits in an AIS rat model [140]. In the same way, the cytotoxic or neuroprotective effects of the modulation of LPS-induced inflammation through TLR4 can be also partly explained by the release of different mediators from glial cells after ischemia insults [141]. It has been reported that expressions of TLR2 and TLR4 in plasma of patients with AIS are associated with poor outcomes and greater inflammation [81].

HMGB1, a key ligand for TLRs, is released from necrotic cells, activates microglia, and induces an early inflammatory response via the NF-kB pathway within the ischemic tissue following AIS [142,143]. HMGB1 has been considered as a potential biomarker for predicting the prognosis of AIS [94]. HMGB1 modulates neurovascular repair and tissue remodeling after AIS and its plasma values correlate with the outcomes of ischemic insult [144,145,146]. In the early phase of AIS, matrix metalloproteinases (MMPs) disrupt the blood-brain barrier (BBB), enhance brain edema and hemorrhage, and aggravate tissue injury [147]. Consequently, Enhanced plasma values of MMP-9 and HMGB1 are correlated with each other in early phases of brain ischemia and associated with a poor functional outcome in patients with AIS [148]. HMGB1 exhibits its pro-inflammatory effects by the activation of TLR2 and TLR4 and consequently the NF-kB pathway [149,150]. Furthermore, HMGB1 release activates TLR-4 and increases IL-1β production through Nod-like receptor protein 3 inflammasome activation [151]. Inactivation of HMGB1 triggers microglia activity and increases neuronal N-methyl-D-aspartic acid-induced injury/death via TLR-4 signaling [152]. Taken together, HMGB1 enhances stem and progenitor cell recruitment, neurogenesis, and angiogenesis in the ischemic brain via the TLR signaling pathway [153,154]. In one study, dexmedetomidine, a centrally acting alpha2-adrenoceptor agonist with sedative and anesthetic properties, alleviated cerebral ischemia injury in a post-conditioning MCAO rat model [155]. Administration of dexmedetomidine led to the reduction in inflammatory responses, oxidative stress insult, infarction area, and brain water content as well as to improve neurological function score in ischemic rats through the downregulation of HMGB1/TLR4/NF-kB pathway [155]. During delayed phases of AIS, HMGB1 may enhance the interaction among various cells and play a beneficial immunomodulatory role in tissue recovery [57].

Furthermore, several studies have indicated the role of TLR7, TLR8, and TLR9 in the greater neuroinflammatory responses as well as poor outcomes in subjects suffering from ischemic insults. Moreover, the expressions of TLR7 and TLR8 were associated with poor outcomes in 110 patients with AIS [111]. Moreover, TLR7 and TLR8 values have interacted with IL1β and IL6 levels, and the expression of TLR8 was associated with cerebral infarct volumes [111]. Activation of TLR7 decreases infarct volume and improves functional impairments via the enhancement of interferons before middle cerebral artery occlusion in mice; implicating the TLR7 preconditioning-mediated neuroprotection against AIS [108]. Application of a TLR7 ligand improves atherosclerotic lesion burden, reduces plasma cholesterol, and promotes B-cell protective property in mice, which can exert an anti-inflammatory effect on the lesion site [156]. *MicroRNA-18a-5p* (*miR-18a-5p*) exerts a neuroprotective effect on oxygen-glucose-deprivation/reoxygenation-induced cell injury of the rat pheochromocytoma PC12 cells. Upregulation of *miR-18a-5p* decreases the expression of TLR4 and TLR7, whereas overexpression of TLR8 overturns the *miR-18a-5p*-induced cell protection [157].

Numerous investigations have revealed that preconditioning with TLR2, TLR4, and TLR9 agonists before the occurrence of AIS results in a greater ischemic tolerance [158]. It has been suggested that the modulation of TLRs could be beneficial in reducing sustained pathological alterations that follow the early AIS damages [159]. Suppression of the TLR2 signaling pathway after mild AIS regulated microglial infiltration and neuronal injury [82,121,160]. TLR4 is a key regulator of axonal debris clearance by microglia, which creates a more suitable environment for axonal outgrowth following neuronal injury [161]. The application of TLR agonists modulates a specific interferon response in microglia, which contributes to preconditioning-mediated neuroprotection against hypoxia-ischemia insults [30]. It has been shown that resveratrol, a naturally occurring polyphenolic compound [162], modulates microglial activity, exhibits neuroprotective effects, and improves stroke outcome through the regulation of TLR4/NF-kB/ signal transducer and activator of transcription 3 (STAT-3) signaling pathway [163,164,165]. Stevioside, a natural sweet-tasting glycoside derived from *Stevia rebaudiana*, exerts a protective effect against permanent cerebral ischemia injury and microglia morphological changes through the inhibition of the TLR/NF-kB pathway-mediated neuroinflammation in an animal stroke model [166]. Moreover, *miR-155* downregulates after a stroke [163,167] that could lead to the improvement of stroke outcomes [168]. The expression of *miR-155* increases by the activation of different TLRs, including TLR2, TLR3, TLR4, and TLR9 [169,170]. In comparison, neurosteroids, such as progesterone and estrogen, may exert their neuroprotective effect on the ischemic brain via the modulation of the TLR signaling pathway [16]. Indeed, the application of progesterone and its metabolites modulate TLR4-NF-kB signaling pathways, reduce inflammation, and prevent neuronal death after AIS, SAH, and ICH in different animal models [171,172,173]. In particular, administration of progesterone and vitamin D improves the outcome of brain ischemia via the modulation of the TLR4/NF-kB signaling pathway in an AIS animal model [174]. The estrogen receptor G protein-coupled receptor-30 provides acute neuroprotection against AIS through the inhibition of TLR4-mediated microglial inflammation following MCAO in mice [175]. Melatonin also exerts its neuroprotective properties via the modulation of TLR4 signaling [153]. Application of TAK-242, a TLR4 antagonist, following the induction of MCAO in diabetic and nondiabetic rats significantly reduced brain edema, decreased hemorrhagic transformation and infarct size, and improved functional outcome [92]. Furthermore, using DNA aptamers (single-stranded oligonucleotides) to block TLR4 revealed a protective role in rat AIS models [93]. Microglial-healing peptide, a recombinant anti-TLR compound, significantly decreased the ischemic injury and tissue plasminogen activator-induced hemorrhage formation in a mice AIS model [91,176]. Administration of Schisandrin B, a compound extracted from *Schisandra Chinensis*, in a rodent rat model of AIS caused a reduction in infarct size and inflammatory mediators, and an improvement in neurological impairments through the downregulation of TLR4 expression in the neocortex [177]. Isoquercetin, a dietary flavonoid, inhibited inflammatory responses, reduced apoptosis and cell injury, and promote neuronal recovery through the inhibition of TLR4 and caspase activity in an in vivo MCAO rat model and an in vitro oxygen-glucose deprivation neuron model [76,83]. In one study, Propofol, a sedative drug, could exert anti-inflammatory properties via the downregulation of TLRs and reduction of pro-inflammatory cytokines [178,179]. The application of propofol significantly reduced infarct volume and pro-inflammatory cytokines in a mouse model of AIS [180]. As a result, the prophylactic application of TLR4 modulators has been suggested as a novel approach for the prevention of neuroinflammation and AIS tissue injury [131,181].

In the same way, macrophage-inducible C-type lectin, an innate immune receptor C-type lectin-like receptor, and its downstream phospho-Syk/Syk (SYK) are upregulated following AIS [124,182]. Therefore, SYK inhibitors were suggested as the potential therapeutic compounds for the treatment of AIS [183]. The activation of TLRs interacts with SYK and leads to the release of various pro-inflammatory cytokines [121]. As a result, administration of piceatannol, an SYK inhibitor, reduced the infarct volume and brain edema after brain ischemia [122,123].

Moreover, TNFα signaling has also a critical role in TLR ligands preconditioning. A high TNF-α plasma value is a risk factor for patients with AIS [184]. Human post-mortem brain tissues obtained from patients with AIS have also shown a marked enhancement of TNF in neurons and glial cells [185]. Similar to other studies, preconditioning with LPS before induction of brain ischemia protects the neuronal tissues against the cytotoxic effects of the TNF-α pathway in mice [72]. In particular, activation of TLRs inhibited cytotoxic TNFα and increased neuroprotective IFN and type I interferons [113,114,116]. Likewise, enhancement of ischemic tolerance following temporary bilateral common carotid artery occlusion in mice was correlated with a significant downregulation of TNF-α in wild-type TLR4 mice compared with knockout mice [186]. Systemic application of the TLR9 ligand cytosine-guanine oligodeoxynucleotides (CpG-OdN) before MCAO-induced brain ischemia significantly decreased ischemic damage through a TNF-dependent process [99] and PI3K/Akt signaling pathway [187]. Modulation of TLR9 also exhibited neuroprotective effects in other animal AIS models [113,114,116].

AIS occurred in 2.5% of patients with TBI and is associated with worse functional outcomes [188]. TLR-mediated inflammatory responses are associated with brain tissue injury and neurological impairments following TBI [76]. The expression of TLR2 and TLR4 significantly increased in macrophages and microglia in lesion sites and in the subcortical white matter following TBI [189]. Knockdown of TLR4 improves TBI-induced inflammatory response and tissue damage via suppressing neuronal autophagy and glial cell activation [190]. Furthermore, knockdown of TLR4 decreases infarct volumes and promotes functional outcomes in a TBI mice model [191].

### 3.2. The Role of TLRs in ICH

ICH occurs when blood vessels in the brain rupture due to various pathological conditions, such as hypertension and microangiopathy [192]. Blood leakage in the surrounding tissue leads to edema, changes in cerebral blow flow (CBF), and neuronal injury with various mechanisms, including activation of inflammatory cascades [193,194]. Both the primary ICH and the surrounded cerebral edema play a critical role in post-hemorrhagic secondary brain injury [195,196]. The potential interactions between microglia, astrocytes, neurons, and oligodendrocytes, as well as microglial phagocytosis, play a decisive role in the progression and outcomes of ICH [197,198]. In this regard, TLRs contribute to the microglial phagocytosis, and subsequent neuroinflammation and tissue injury following ICH [199,200]. A greater expression of TLR2 and TLR4 in monocytes and neutrophils in patients with ICH is accompanied by a larger lesion volume and grave outcomes [85]. Moreover, induction of ICH in TLR4 knock-out mice led to significant alleviation of peri-hematomal inflammation, the decrement in inflammatory cells, and consequently improvement of functional outcomes [201].

Application of TLR4 antagonist TAK-242 led to a significant improvement of neurological functions and reduction of brain edema, peripheral inflammatory cell infiltration in an ICH mouse model through the downregulation of TLR4 and its downstream molecules NF-kB, MyD88, and TRIF [96]. Modulation of TLR-mediated signaling pathway may alter the level of ICH-induced inflammation and brain tissue damage. Antcin C, a steroid-like substance isolated from *Antrodia cinnamomea*, improved neurological deficits and reduced cerebral injury in an ICH experimental model through the inhibition of the TLR4 pathway and microglia-induced inflammation [95]. Cinnamaldehyde, a diterpene with strong anti-inflammatory properties, improved neurological functions, decreased brain edema and infarct size, suppressed the activation of TLR4, TNF, and NF-kB, and reduced leukocyte infiltration in mice model of AIS [98]. Luteolin, a common type of catechol-type flavonoid, reduced cytokine release, neuroinflammation, and neurological functional impairments by antagonizing TLR4/NF-kB signaling pathway in an ICH rat model [97]. Verbascoside, a phenylpropanoid glycoside, also inhibited the TLR4/NF-kB pathway and significantly decreased microglial activation, inflammatory mediators, brain edema, hematoma volume, and neuronal apoptosis in an experimental model of ICH [94]. Furthermore, the application of TLR7 agonist imiquimod increased heme scavenging following ICH induced by intrastriatal injection of collagenase in mice [109]. In addition, the value of TLRs may predict the prognosis of ICH. More Specifically, Greater TLR7 values in atherosclerotic plaques removed by carotid endarterectomy were associated with a better outcome with a lower risk of cerebrovascular complications in an 8-year follow-up study in 123 patients [202].

The release of Iron into the brain’s parenchyma after ICH leads to the enhancement of pro-inflammatory mediators, which is associated with poor prognosis [203]. Heme oxygenase deficient mice exhibit a significant reduction in reactive oxygen species production, microglia activation, and leukocyte infiltration after ICH [198]. Moreover, heme iron activates TLRs-NF-kB signaling, triggers microglia-induced neuroinflammation, and worsens neurological impairments in an experimental model of ICH [17,89,96]. Furthermore, heme-induced inflammation activates microglia, which leads to further release of pro-inflammatory mediators via the assembly of TLR2/TLR4 heterodimers in a MyD88-dependent pathway [204]. The release of heme to the neural tissues following ICH activates TLR2 in astrocytes, enhances neuroinflammatory gene expression, disrupts BBB, and increases brain injury [84,205]. Activation of TLRs enhanced the expression of hepcidin, a key regulator of iron homeostasis, by upregulating IL6 expression and STAT-3 pathway in a mice ICH model. Application of TAK-242 improved brain iron efflux, diminished oxidative injury, and improved cognitive performance in these mice [206]. Serum values of cold-inducible RNA-binding protein, a cold shock protein, were significantly increased in the patients with ICH. Induction of ICH in CIRP-deficient mice led to lower expressions of TLR4 as well as fewer neurological impairments and inflammatory marker levels compared to the control mice [207].

### 3.3. The Role of TLRs in SAH

Numerous studies focused mainly on vasospasm as the central mechanism for the delayed neurological impairments in SAH. However, the post-SAH delayed neurological deficits can occur regardless of vasospasm, and therefore, the other mechanisms, such as impaired cerebral autoregulation, microthromboembolism, cerebral edema, CBF loss, spreading depression, and neuroinflammation may play a role in triggering the delayed neurological deficit after SAH [208]. Evidence from experimental and clinical studies suggests the possible involvement of TLRs in both vasospasm and direct neuronal tissue injury as well as in hypoxia due to impaired CBF flow [209,210]. TLR-mediated inflammation plays a pivotal role in SAH [211,212]. Several experimental investigations have shown an up-regulation of the TLR2/4-MyD88-NF-κB signaling pathway in early brain injury following SAH [213,214,215]. Preconditioning with LPS resulted in a decreased number of microglia expressing TLR4 on their surface in a mice model of SAH [216]. It has been suggested that TLR4 antagonists with appropriate BBB penetration could be the potential therapeutic candidates for SAH [217]. Upregulation of TLRs after SAH, which was correlated with SAH-induced early brain injury, has been observed in a rodent SAH model [86]. Besides, significant correlations have been observed between the up-regulation of TLR4 and the occurrence of cerebral vasospasm after SAH in animal studies [218]. In particular, enhancement of the expression of TLR4 on the endothelial cell layer of human cerebral aneurysms has proposed the possible role of TLR4 in the formation of brain aneurysms [219,220]. Inhibition of the TLR-TRIF signaling pathway decreases vascular inflammation and protects against angiotensin II-induced aneurysm formation in apolipoprotein E-deficient mice [221]. TLR4 depletion decreased the levels of pro-inflammatory cytokines and ameliorated neurological deficit in a mice model of SAH [100]. Extravascular hemolysis and the release of heme after SAH exert a toxic effect on membrane homeostasis, which can be resulted in inflammation, neuroglial dysfunction, and cell death [222]. Heme acts as a potent activator of TLR4 as well as the MyD88 and TRIF cascades that lead to neuroinflammation [204]. This effect may be mediated through the activation of heme oxygenase-1, an enzyme that catalyzes the degradation of heme [223]. While the activation of the TLR4-MyD88 cascade is mandatory for tissue injury and vasospasm in the early stage of SAH, tissue injury and vasospasm in the late phase of SAH are mediated through the TLR4-TRIF pathway [99]. In addition, a part of the detrimental effect of TLR4 signaling following SAH could be mediated by the activation of microglial cells [224]. In contrast, the depletion of microglia alleviates TLR4-induced inflammation and both the early and late phases of brain injury following SAH [99]. Besides, the TLR4 signaling pathway contributes to the intracranial aneurysmal rupture through the enhancement of inflammation in aneurysmal walls. Knockout of macrophage/monocyte-specific TLR4, as well as the deficiency of MyD88, significantly reduced the aneurysmal rupture rate in a mouse model of the intracranial aneurysm [141]. A significant upregulation of the TLR2 gene and greater expression of TLR2 protein in ruptured aneurysms has been observed in patients with intracranial aneurysms [225,226]. It has been suggested that the enhancement of TNF-α expression via the activation of TLRs, particularly TLR2 and TLR6, in aneurysm walls may contribute to neurological deficits after SAH [227].

Several substances exert their anti-inflammatory and neuroprotective effects on SAH-induced brain injury via the modulation of TLRs. Tenascin-C, an extracellular matrix glycoprotein, triggers a severe cerebral arterial contraction following SAH [28]. TLR4 activation induces tenascin-C and administration of tenascin-C increases the TLR4 expression in the smooth muscle layer of the affected brain arteries. This process leads to the enhancement of both systemic and subarachnoid inflammatory responses [28]. Application of TLR4 antagonist lipopolysaccharide from *Rhodobacter sphaeroides* (LPS-RS) inhibits tenascin-C-mediated cerebral vasospasm in rats [228,229]. Furthermore, the activation of the TLR4-tenascin-C pathway is implicated in the BBB disruption, cell damage and apoptosis, and cerebral vasospasm following SAH in animal experimental models [28]. Treatment with tenascin-C resulted in enhancement of TLR4, JNK, and p38 in the smooth muscle cell layer of the spastic brain arteries. These effects were inhibited by the application of TLR4 selective antagonist LPS-RS in rats [228]. Likewise, functional and numbers abnormalities of circulating myeloid and plasmacytoid dendritic cells in patients suffering from SAH have been reported. TLR-3 and 4 stimulation as well as TLR-3 agonist poly I:C markedly reduced the frequency of myeloid dendritic cells producing cytokines [117]. The delayed cerebral ischemia after SAH is associated with greater differentiation of monocytes into antigen-presenting plasmacytoid dendritic cells [230]. Plasma values of galectin-3, a member of the lectin family and a matricellular protein, increased following SAH that resulted in greater inflammatory responses and contributes to poor outcomes in subjects with SAH [231]. Administration of galectin-3 inhibitor prevents the BBB disruption, presumably via binding to TLR4 and the activation of ERK1/2, STAT-3 pathway [102]. Pentoxifylline, a *xanthine* derivative and a non-selective phosphodiesterase inhibitor, exerts anti-inflammatory and neuroprotective effects on early brain injury after SAH, which is associated with a significant reduction in the expressions of TLR4, NF-kB, MyD88, and the downstream pro-inflammatory mediators [103]. The natural flavonoid fisetin exhibits anti-inflammatory and protective properties on SAH-induced neuronal tissue injury, probably via the suppression of the TLR4/NF-kB-mediated signaling pathway. Fisetin significantly decreased the expressions of TLR4 and NF-kB, reduced the values of pro-inflammatory cytokines, and inhibited neural cell apoptosis following SAH in an experimental rodent model [104]. The purine antimetabolite 6-mercaptopurine (6-MP), a hypoxanthine analog, exerts an anti-apoptotic property on SAH-induced early brain injury through the inhibition of TLR2 and TLR4 expressions [86]. Curcumin, a plant-derived polyphenolic substance, alleviated SAH-induced neuroinflammation via the suppression of the TLR4/MyD88/NF-kB signaling pathway [100]. Furthermore, the application of fluoxetine, a serotonin selective reuptake inhibitor, attenuated neuronal injury, decreased the number of microglia, reduced inflammatory responses, and promoted neurological deficits after SAH via the inhibition of TLR4/MyD88/NF-kB signaling pathway in rats [101].

On the other hand, the manipulation of TLRs improves the SAH outcome in experimental studies. The inhibition of TLR4 with selective antagonist blockers improved SAH-induced of BBB disruption in a SAH animal model [232,233]. Application of TLR4 inhibitors IAXO-102 and TAK-242 in a rodent model of SAH significantly decreased SAH-induced neurological impairments, the BBB dysfunction, and subsequent brain edema [233,234,235]. SAH led to TLR4 activation and COX-1 upregulation in the smooth muscle and endothelial cells of the constricted cerebral arteries in mice. Administration of two selective TLR4 inhibitors IAXO-102 and LPS-RS prevented cerebral vasospasm and neurological impairments following the induction of SAH in these mice [236]. Furthermore, the application of the TLR4 antagonist TAK-242 or the MyD88 inhibitor ST2825 considerably lessened the neural tissue injury and inflammatory responses produced by sleep deprivation following SAH in rats [237].

Regulation of peroxisome proliferator-activated receptor-gamma (PPAR-γ) signaling pathway plays a crucial role in the protection of neural cell functions after SAH [238]. Activation of PPAR-γ receptors potently inhibits the expression and activity of TLR4 [239]. Rosiglitazone, a PPAR-γ agonist, reduced the SAH-induced tissue injury and cerebral vasoconstriction in basilar arteries through the inhibition of the TLR4 signaling in rats [105]. Rosiglitazone also inhibited oxyhemoglobin-induced TLR4 activation and cytokine release in cultured vascular smooth muscle cells through the activation of PPAR-γ [42]. Moreover, pannexin-1 channels, a transmembrane protein of gap junction channels, regulate vessel function and permeability through releasing of signaling molecules, such as ATP, and contribute to both the innate and acquired immune systems via the modulation of cytokine values [240,241]. Pannexin-1 channels protein gene knockdown remarkably suppress the TLR2/4/NF-kB pathway and promoted cognitive functions in an experimental model of SAH [242].

### 3.4. The Role of TLRs in CVST

Enhancement of cytokine release following inflammatory responses increases the risk of venous thrombosis [243]. It has been suggested that inflammation activates TLRs and leads to venous thrombosis. Inflammatory mediators released during hypoxia and impaired venous flow activate TLR3, which further upregulates the expression of fibrin deposition via the TLR3-ERK1/Activator Protein-1 pathway and increases the risk of venous thrombosis [6]. Furthermore, the activation of the TLR-4 pathway leads to late activation of NF-kB and the toxic reaction of TNF-α, which consequently enhances the risk of thrombosis [106,107]. TLRs expressed on macrophages are key regulators of cholesterol accumulation and immune responses within the atherosclerotic plaque [244]. The activation of TLR4 and consequently the release of pro-inflammatory mediators, such as TNF-α and IL6, lead to the activation of monocytes, facilitation of transendothelial migration of monocytes into the plaque microenvironment, and contribute to plaque progression and rupture [245]. The potential role of the TLR9 signaling pathway in venous thrombosis was assessed in a rodent model of CVST. Genetic deletion as of TLR9 well as exogenous inhibition of TLR9 leads to the impairment of thrombus resolution and enhanced leukocyte infiltration in mice [246]. Antiphospholipid syndrome, an autoimmune disease characterized by a hypercoagulable condition, is accompanied by a significant increase in peripheral mononuclear cell expression of both TLR2 and TLR4 and the activation of MyD88 and NF-kB pathways [87,247]. Activation of TLR8 in subjects with the antiphospholipid syndrome has been suggested to contribute to arterial and/or venous thrombosis through the enhancement of TNFα values and consequent inflammatory responses [112].

## 4. Conclusions

TLRs and downstream signaling pathways play a pivotal role in the inflammatory response to tissue injury and its clinical consequences of various CVDs. Due to the dual role of TLRs-induced inflammatory responses following CVDs—beneficial in the initial stage and detrimental in the late phase- appropriate targeting of TLRs could be a promising approach for the prevention and treatment of CVDs. Various substances are available for modulation of each TLR. Whether these compounds exert clinical benefits in patients with CVDs, further experimental and clinical evaluations are required [248].

## Figures and Tables

**Table 1:** A summary of the implications of various Toll-like receptors (TLRs) in different cerebral vascular diseases (CVDs).

CVDs TLRs	AIS	ICH	SAH	CVST
TLR2	TLR2–CD36 complex could act as a sensor of ischemia [25] Associated with poor outcome and greater inflammation in patients with AIS [81] Regulation of leucocytes and microglial infiltration and neuronal injury/death [82,83]	Hb-induced TLR2/TLR4 heterodimer modulates inflammation following ICH [84] TLR2 expression in immune cells is correlated with lesion size in ICH patients [85]	Anti-inflammatory effects through suppression of the TLR2-mediated signaling pathway [86]	Activated in antiphospholipid syndrome [87]
TLR3	Neuroprotective and anti-inflammatory effects on AIS-induced neuroinflammation [51] Enhancement of ischemic tolerance by TLR3 ligand poly:IC preconditioning via IFN type I [88]	-	-	Activation leads to the upregulation of the fibrin deposition and increases the risk of venous thrombosis [6]
TLR4	LPS-induced neuroprotective effect by preconditioning through inhibition of NF-kB activity, enhancement of IRF3 activity and anti-inflammatory IFN gene expression [82,83,89] Induction and evolution of atherosclerosis through NF-kB pathway that produces inflammation [82,83] LPS-induced neuroprotective effect by preconditioning with propranolol, a nonselective β-adrenergic receptor blocker [90,91] Reduction in brain edema, hemorrhagic transformation, and infarct size and improvement in functional outcome by TAK-242 and DNA aptamers, TLR4 antagonists [92,93]	Activation leads to enhanced values of inflammatory factors, DNA damage, and neuronal degeneration in the peri-hematomal region [89] Inhibition results in decreasing microglial activation, inflammatory mediators, brain edema, hematoma volume, and functional impairment [94,95,96,97,98]	Activation by Heme product through the MyD88 and TRIF pathways [99] anti-inflammatory and neuroprotective effects via the suppression of the TLR4/NF-kB-mediated signaling pathway by galectin-3 inhibitor, pentoxifylline, fisetin, Curcumin, 6-mercaptopurine, Rosiglitazone and fluoxetine [86,100,101,102,103,104,105]	Activated in antiphospholipid syndrome [87] Activation of the TLR-4/NF-kB pathway leads to the toxic reaction of TNF-α and enhances the risk of thrombosis [106,107]
TLR7	Increasing ischemic tolerance by preconditioning with a TLR7 ligand, decreasing infarct volume, improving clinical outcome via IFN type I [108]	Facilitates heme scavenging by modulating the BTK-CRT-LRP1-Hx pathway [109]	-	TLR7 plays a role in procoagulant production in post-infection inflammatory responses [110]
TLR8	Activation leads to increasing brain injury following events [111]	-	-	Activated in antiphospholipid syndrome [112]
TLR9	Increasing ischemic tolerance by TLR9 ligand preconditioning via inhibition of cytotoxic TNFα, enhancing IRFs and generation of IFN type I [29] leading to lower ischemic damages [113,114,115,116]	-	Decreased ability of TLR9-induced IFN-α secretion [117]	Deletion of TLR9 resulting in greater venous thrombosis and enhancing leucocyte infiltration [87]

Abbreviations: AIS: Acute Ischemic Stroke; ICH: Intracranial Hemorrhage; SAH: Subarachnoid Hemorrhage; CVST: Cerebral Venous Sinus Thrombosis; APS: Antiphospholipid Syndrome; TNF: Tissue Necrosis Factor; IFNAR: The interferon-α/β receptor.

## Data Availability

Not applicable.
